# An Unusual Presentation of Propylthiouracil-Induced Immunoglobulin A Vasculitis With Positive Anti-proteinase-3 and Anti-myeloperoxidase Antibodies

**DOI:** 10.7759/cureus.58535

**Published:** 2024-04-18

**Authors:** Michelle K Custer, Trey Kidd, Kate Ducker, Rekha Lall

**Affiliations:** 1 Dermatology, Edward Via College of Osteopathic Medicine, Auburn, USA; 2 Internal Medicine, East Alabama Medical Center, Opelika, USA; 3 Internal Medicine, Edward Via College of Osteopathic Medicine, Auburn, USA

**Keywords:** drug-induced vasculitis, anca-associated vasculitis, propylthiouracil, hyperthyroidism, iga vasculitis

## Abstract

Propylthiouracil (PTU) has been identified as a known cause of anti-neutrophil cytoplasmic antibodies-associated vasculitis. However, the association between PTU and immunoglobulin A (IgA) vasculitis remains uncertain due to its rarity and diverse clinical presentation. Here, we report the case of a 57-year-old female with a past medical history of chronic leukopenia and Graves’ disease treated with PTU that presented with pancytopenia and widespread non-blanching ecchymoses on the bilateral legs. A punch biopsy of the medial leg demonstrated IgA vasculitis and autoimmune antibody analysis revealed increased levels of anti-proteinase 3 antibodies compared to anti-myeloperoxidase antibodies. These findings led to the diagnosis of PTU-induced IgA vasculitis. Following the discontinuation of PTU, there was marked improvement in the appearance of the patient’s cutaneous manifestations and hematological indices.

## Introduction

Graves’ disease is an autoimmune disorder associated with the production of anti-thyroid-stimulating hormone receptor autoantibodies that stimulate thyroid follicular cells within the thyroid gland, leading to an amplified secretion of thyroid hormones [1]. This excess production of thyroid hormones results in hyperthyroidism, presenting with tachycardia, palpitations, weight loss, heat intolerance, and extrathyroidal manifestations such as exophthalmos [1]. Hyperthyroidism most commonly arises from Graves’ disease, and its development relies on genetic and environmental factors [1]. Treatment mainstays for Graves’ hyperthyroidism include thioamide medications, radioactive iodine, and surgery [2]. The selection of therapy for Graves’ disease is tailored to the patient [2].

Propylthiouracil (PTU) is an antithyroid medication that is associated with adverse reactions, including drug-induced hematopoietic changes such as agranulocytosis and pancytopenia [3]. Additionally, drug-induced autoimmune syndromes associated with PTU usage include drug-induced lupus or drug-induced vasculitis [4]. Regarding vasculitis, PTU has been identified as a known cause of anti-neutrophil cytoplasmic antibodies (ANCA)-associated vasculitis [5]. The presence of anti-myeloperoxidase (MPO) antibodies has been observed to be more prevalent than anti-proteinase 3 (PR3) antibodies in ANCA-associated vasculitis [5]. Most patients with observable levels of ANCA on serology will not display presenting features of vasculitis such as palpable purpura, arthralgia, renal impairment, or respiratory tract involvement [5].

Immunoglobulin A (IgA) vasculitis is an IgA-mediated disease that mainly affects small blood vessels and occurs less frequently in adults [6]. IgA vasculitis presents with purpura, arthralgias, inflammatory bowel disease, and renal dysfunction [6,7]. Literature analysis revealed a case of IgA vasculitis as an adverse reaction to PTU overdose [8]. However, there remains an unclear understanding of the relationship between PTU and IgA vasculitis [9].

We present a case of PTU-induced IgA vasculitis characterized by elevated levels of anti-PR3 antibodies, which were higher than anti-MPO antibodies. We also address the importance of a timely diagnosis and implementation of appropriate therapies to prevent further disease progression and reduce disease burden.

## Case presentation

A 57-year-old female with a history of chronic leukopenia and Graves’ disease managed with PTU presented to the emergency department with two months of ongoing weakness and dyspnea. On admission, the patient displayed symptomatic anemia, and a laboratory workup revealed pancytopenia, as shown in Table 1. Additionally, her T4 level was 0.47 μg/dL and her thyroid-stimulating hormone level was 10.63 μU/mL, which is the most hypothyroid she had been during treatment of her disease. With a clinical suspicion of iatrogenic hypothyroidism and for potential association with pancytopenia, PTU was held until further workup was completed. Blood smear, bone marrow biopsy, cytogenetics, and flow cytometry were unremarkable. Following an improvement in hematological parameters subsequent to holding PTU, the patient’s pancytopenia was believed to be provoked by PTU. The patient was instructed to discontinue PTU and was scheduled for a two-week follow-up.

**Table 1:** Complete blood count results on admission to the emergency department. MCH: mean corpuscular hemoglobin; MCV: mean corpuscular volume; PLT: platelets; RBC: red blood cells; RDW: red cell distribution width; WBC: white blood cells; ESR: erythrocyte sedimentation rate; CRP: C-reactive protein

Laboratory test	Result	Reference range
Hematocrit	25.6%	36–46%
Hemoglobin	8.5 g/dL	12.0–16.0 g/dL
MCH	26.5 pg/cell	25–35 pg/cell
MCV	81.7 fL	80–100 fL
PLT	121 × 10^3^/µL	150–400 × 10^3^/µL
RBC	2.8 × 10^6^/µL	3.5–5.5 × 10^6^/µL
RDW	14.8%	12–15%
WBC	0.6 × 10^3^/µL	4.5–11 × 10^3^/µL
ESR	33 mm/hour	0–20 mm/hour
CRP	1.6 mg/dL	0–0.9 mg/dL

**Figure 1:**
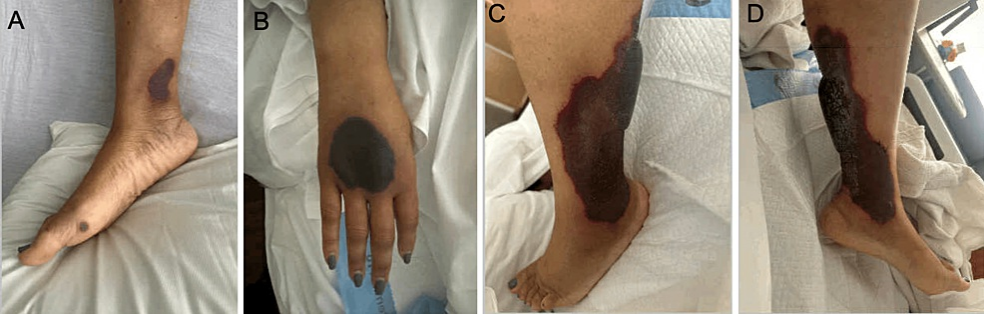
Patient’s dermatological manifestations. (A) Ecchymosis present on the right medial malleolus and purpuric lesion located on the distal lateral aspect of the right foot. (B) Ecchymosis present on the patient’s anterior right hand. (C) Left leg displaying prominent ecchymosis. (D) Left leg displaying prominent bullae.

On follow-up, the patient endorsed continuing fatigue and presented with four days of ongoing bilateral leg edema, cramping, and pain with no known inciting factor. The following day, the patient noted increased pain and developed extensive non-blanching ecchymosis and bullae with palpable purpura on the bilateral legs and anterior surface of the right hand, as noted in Figure 1. The right auricle and malar region of the face also displayed hyperpigmentation. The active range of motion was decreased in the right hand. The patient denied fever, chills, nausea, vomiting, or a history of a bleeding disorder.

**Figure 2:**
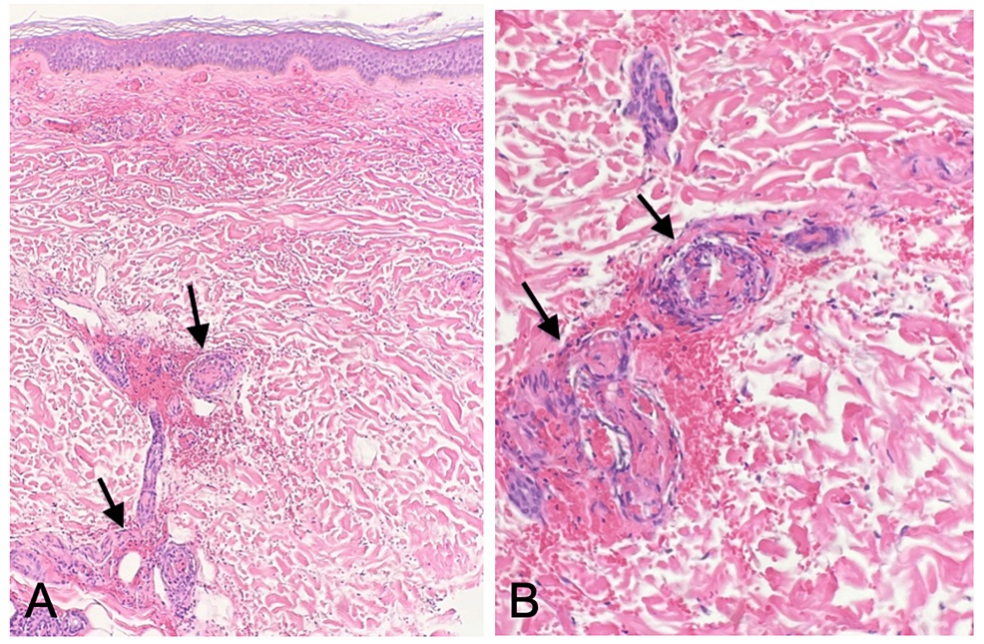
Skin biopsy demonstrating small vessel vasculitis. (A) 100× image of the skin biopsy with hematoxylin and eosin (H&E) stain showing infiltration of inflammatory cells and extravasation of erythrocytes. (B) 400× image of the skin biopsy with H&E stain showing predominant neutrophilic inflammation and erythrocyte extravasation.

**Figure 3:**
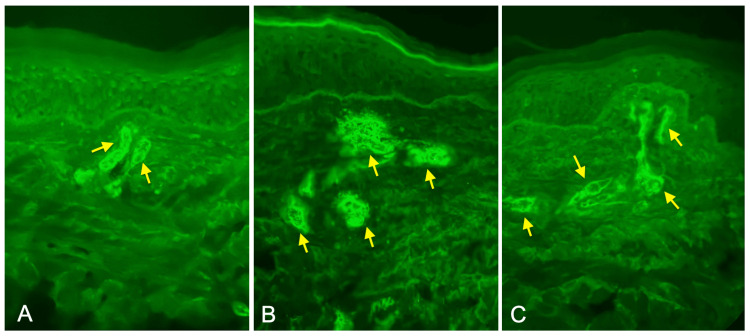
Skin biopsy displaying direct immunofluorescent staining of (A) C3, (B) fibrinogen, and (C) IgA. Direct immunofluorescent staining of skin biopsy demonstrating (A) complement component 3 (C3), (B) fibrinogen, and (C) immunoglobulin A (IgA) deposition with the patient’s tissue.

Lower extremity venous duplex ultrasound ruled out deep vein thrombosis but revealed incidental occlusive superficial thrombophlebitis of the distal greater saphenous vein. Chest X-ray and computed tomography (CT) with contrast showed no signs of vasculitis. The patient underwent a 4 mm punch biopsy of the left medial leg and direct immunofluorescence staining, which revealed vascular deposition of IgA, C3, fibrinogen, and focal IgM resulting in a diagnosis of IgA vasculitis with thromboembolic vasculopathy, as shown in Figure 2 and Figure 3.

As displayed in Table 2, autoimmune serologies revealed a positive anti-nuclear antibody titer, weakly positive anti-histone antibodies, and negative anti-dsDNA antibodies. Levels of IgA and IgG were elevated at 477 U/mL and 2,210 U/mL, respectively. Autoimmune serology revealed that both anti-PR3 and anti-MPO antibodies were disproportionately elevated with higher levels of anti-PR3 antibodies. Given the results of the autoimmune serologies, skin biopsy, and the patient’s prior PTU usage, PTU-induced IgA vasculitis was suspected. The patient’s treatment consisted of an oral steroid taper with an initial dose of 20 mg of prednisone for two weeks, followed by 10 mg of prednisone for two weeks, and finally 5 mg of prednisone for an additional two weeks.

**Table 2:** Patient’s autoimmune serologies. Ab: antibody; dsDNA: double-stranded DNA; IgG: immunoglobulin G; IgM: immunoglobulin M; IgA: immunoglobulin A; ANA: anti-nuclear antibody; PR3: proteinase 3; MPO: myeloperoxidase

Autoantibody	Result	Reference range
Anti-dsDNA	1 U/mL	<1 U/mL
Anti-histone Ab	1.1 U/mL	<1 U/mL
Beta-2-glycoprotein IgG Ab	<2 U/mL	<20 U/mL
Beta-2-glycoprotein IgM Ab	<2 U/mL	<20 U/mL
Cardiolipin IgG Ab	<2 U/mL	<20 U/mL
Cardiolipin IgM Ab	<2 U/mL	<20 U/mL
Cyclic citrulline peptide	<16 U/mL	<20 U/mL
ANA titer	>1:1,280	Negative
Immunoglobulin A	478 U/mL	47–310 U/mL
PR3-Ab	54.7 U/mL	<1 U/mL
MPO-Ab	5.5 U/mL	<1 U/mL

On follow-up, there was a significant reduction in pain and edema with improvement in the appearance of ecchymoses. The patient underwent excisional debridement for vasculitic ulcers of the left leg, right ankle, and right hand. The patient intends to follow up with endocrinology and rheumatology for further disease management.

## Discussion

In this case, we presented a patient with suspected PTU-induced IgA vasculitis with elevated levels of anti-PR3 when compared to anti-MPO antibodies. PTU-induced vasculitis is a known adverse reaction associated with thioamide medications used in the treatment of Graves’ disease. The clinical symptoms of PTU-induced vasculitis may include cutaneous hyperpigmentation, ecchymoses, purpura, lesions, and joint, renal, or pulmonary involvement with a difference in severity among patients [10]. Although the association of PTU-induced ANCA vasculitis has been well-documented, the association of PTU-induced IgA vasculitis remains unclear and possibly underreported [9]. Regarding PTU-induced ANCA vasculitis, the presence of a higher level of anti-MPO antibodies compared to anti-PR3 antibodies has been the more common presentation of this adverse reaction. However, this patient displayed higher levels of anti-PR3 antibodies [11]. This differentiates our case and provides insight into a unique presentation, further emphasizing the challenges in diagnosis and treatment.

Due to an atypical presentation, the management of this patient posed several challenges. Differential diagnoses included hypersensitivity vasculitis, drug-induced vasculitis, and systemic lupus erythematosus. Initially, due to our clinical suspicion of iatrogenic hypothyroidism, the patient’s original PTU dose of 50 mg three times a day was changed to 25 mg three times a day. Before initiating the new dose, the patient’s PTU was temporarily held during diagnostic evaluation to eliminate potential factors that might have contributed to her pancytopenia. Following an unremarkable laboratory analysis and a few days of hospitalization, her pancytopenia improved, which led to the suspicion of PTU as the cause of her pancytopenia and prompted the decision to discontinue her PTU.

In response to the patient’s ongoing edema, cramping, and pain on follow-up, a 4 mg oral methylprednisolone dose pack was administered. However, due to the progression of her symptoms, the patient transitioned to therapeutic treatment with intravenous solumedrol 80 mg 2 mL every eight hours, resulting in an improvement in clinical symptoms. Because imaging revealed occlusive vasculopathy, the patient was administered apixaban. The clinical indicators that led to the diagnosis of PTU-induced IgA vasculitis included the results of the autoimmune serologies, skin biopsy, and improvement of the patient’s symptoms following discontinuation of PTU. Following the diagnosis of PTU-induced IgA vasculitis, the patient shifted from intravenous steroids to an oral steroid taper.

In addition to PTU-induced IgA vasculitis, as there were concerns about an underlying connective tissue disease due to the patient’s autoimmune serology and distribution of dermatological skin lesions, the patient was started on hydroxychloroquine. The development of Graves’ disease is multifactorial [1]. Patients diagnosed with thyroid disease may be prone to other autoimmune disorders due to shared genetic and environmental triggers [1]. Consequently, patients with Graves’ disease may be at risk for developing additional drug-induced autoimmune disorders such as drug-induced lupus [12]. PTU-induced vasculitis shares clinical characteristics and diagnostic features with other conditions of autoimmunity such as systemic lupus erythematous [12].

## Conclusions

Although PTU is commonly utilized as a first-line pharmacological agent in the management of hyperthyroidism, it has a widely recognized rare adverse effect of ANCA-associated vasculitis. However, the relationship between PTU usage and IgA vasculitis remains incompletely understood, emphasizing the need for further research. Clinical symptoms of PTU-induced vasculitis may include dermatological manifestations, joint involvement, and internal organ impairment. Due to the infrequency and varying clinical appearance of PTU-induced IgA vasculitis, determining diagnostic and treatment modalities may present challenges. Therefore, an interdisciplinary approach is crucial to manage this disease, which was exemplified in the treatment of our patient. Following clinical suspicion for PTU-induced IgA vasculitis, prompt initiation of treatment is critical to reduce further disease progression.
